# Global Optimization Ensemble Model for Classification Methods

**DOI:** 10.1155/2014/313164

**Published:** 2014-04-27

**Authors:** Hina Anwar, Usman Qamar, Abdul Wahab Muzaffar Qureshi

**Affiliations:** Department of Computer Engineering, College of Electrical & Mechanical Engineering (E&ME), National University of Sciences and Technology (NUST), H-12, Islamabad 46000, Pakistan

## Abstract

Supervised learning is the process of data mining for deducing rules from training datasets. A broad array of supervised learning algorithms exists, every one of them with its own advantages and drawbacks. There are some basic issues that affect the accuracy of classifier while solving a supervised learning problem, like bias-variance tradeoff, dimensionality of input space, and noise in the input data space. All these problems affect the accuracy of classifier and are the reason that there is no global optimal method for classification. There is not any generalized improvement method that can increase the accuracy of any classifier while addressing all the problems stated above. This paper proposes a global optimization ensemble model for classification methods (GMC) that can improve the overall accuracy for supervised learning problems. The experimental results on various public datasets showed that the proposed model improved the accuracy of the classification models from 1% to 30% depending upon the algorithm complexity.

## 1. Introduction

According to Han and Kamber [1], “Data Mining is known to be a part of knowledge discovery (KDD) process in which data is analysed and summarized from different perspectives and converted into useful information. It helps in extracting the hidden and valid data which has the potential of being transformed into useful information.” It is similar to machine learning process and can also be termed as supervised learning process. Supervised learning is the process of data mining for deducing rules from training datasets. A broad array of supervised learning algorithms exists, every one of them with its own advantages and drawbacks.

In classification the first step is to divide the data in two portions known as training set and testing set [2]. In these datasets, one attribute must be necessarily defined as class. According to Han et al. [2], the two steps of the classification task are model construction and model usage. In this task, the model is built with the help of trained dataset and then this trained model is used to allocate the unseen records as precisely as possible. While training dataset is used to build and train the model, the testing dataset is used to validate and test the model accuracy [3], which bring us to some of the basic issues that affect the accuracy of a classifier while solving a supervised learning problem. For instance, the bias-variance tradeoff, the dimensionality curse, or the noise in the dataset all contribute towards a decreasing accuracy. Bias arises when the classifier cannot represent the true function; that is, the classifier under fits the data; that is, when it is training on any data set than for a specific input value it is methodically inaccurate when predicting the right outcome for that input value. In contrast to this, variance occurs when the algorithm over fits the data and for a specific input value in a dataset it gives a different outcome every time the training dataset is changed. Another problem that can affect the accuracy of a classifier is the dimensionality or the number of attributes or features in a dataset. If we input a large number of attributes in a classification algorithm even for problems where decision depends on subset of all those attributes, then performance of the classifier will be clouded by high variance due to high dimension of dataset. Therefore if a dataset with high dimension is being used the classifier must make a tradeoff between high bias and low variance. The classification results are also altered by the noise in data, that is, redundant records, incorrect records, missing records, outliers, and so forth. All these problems affect the accuracy of a classifier. Usually the improvements done in a classifier or ensemble model are limited to a very narrow spectrum and they cannot be applied to another classifier under the same conditions.

Classification accuracy is normally improved through ensemble models like bagging (which averages the prediction of a number of classification models), boosting (it uses the voting scheme over a number of classification models), or a combination of classifiers from different or same families as discussed in Section 2.

Therefore, in this paper we propose a global optimization using the idea of ensemble models for classification methods and prove through experimental results that our model improves the classification accuracy of various classifiers on various different public datasets. Section 2 of the paper gives an insight into the previously related work. Design of the proposed model is given in Section 3.  Section 4 explains the implementation.  Section 5 gives the result and analysis.  Section 6 contains the conclusion and future work.

## 2. Literature Review

As mentioned earlier that so far no global optimization ensemble model is present which can help in improving the classification and prediction accuracy for supervised learning problems which are generally affected by a spectrum of issues like dimensionality, accuracy rate, data quality, and so forth. Although, no global solution exists for these problems but some other efforts have been made to resolve these issues and all of them are either algorithm specific or data specific. Every approach has tackled the problem of classification accuracy rate from a different angle and perspective. One such work is [16] where Dash et al. have proved through comparison of various classification techniques like support vector machine (SVM) with polynomial kernel, support vector machine with RBF kernel, radial basis function network (RBFN), and multilayer perceptron network (MLP) with and without feature extraction. It was found that for construction of high performance classification model for microarray dataset, partial least square (PLS) regression method is the suitable feature selection method instead of hybrid dimensionality reduction scheme and feature selection combined with various classification techniques can yield better results. Lin and Chen in [17] combined PSO- (particle swarm optimization-)based approach with commonly used classification technique LDA (linear discriminant analysis). This research also emphasizes the importance of feature selection and its positive effect on classification accuracy. Authors of this study have compared the performance of this combined model called PSOLDA with many other feature selection techniques like forward selection, back propagation selection, and so forth and shown through experimental results that for many public datasets the proposed combined model (PSOLDA) has higher classification accuracy rate. Bryll et al. [18] developed a new wrapper method AB (attribute bagging) to improve the classification accuracy implementing a two-stage method in which first a suitable size was provided for training data and then randomly a subset of attributes was selected for voting scheme. This method was compared with bagging which was used with some decision tree algorithms and some rule induction algorithms, and it was found that the AB performs better in terms of accuracy and constancy. And authors conclude that attribute partitioning is better than data partitioning for improving the accuracy in an ensemble method. Abbott [19] compared boosting with an ensemble of models across the algorithm families. These combined models used voting as the selection scheme and authors report that boosting performs better because it focuses on complicated cases in data and takes into account the confidence value of a particular classification decision. Sohn and Lee [20] tried to improve the classification accuracy of algorithms like neural network and decision trees by applying different approaches including bagging, boosting, and clustering. However for the particular problem of road traffic accident classification clustering leading to classification was found to be more effective. Smith and Martinez [21] suggested that outliers and noise should be eliminated from the dataset as it will yield better results in terms of classification accuracy. Because by removing or filtering these instances the dataset becomes clean of all the cases that could be misclassified. As there is no general definition or guide available as to what noise is and what an outlier is, therefore the identification of these two elements in any dataset is difficult. Furthermore PRISM was found to be one of the best algorithms for finding cases that could be outliers. Dimensionality reduction problem has been an interesting topic for researchers in a diverse spectrum of fields like image detection, voice detection, microarrays, neural network patterns, and so forth. As discussed by Zamalloayz et al. [22], Liu et al. [23], and Raymer et al. [24] genetic algorithm (GA) is quite a popular method under research and is found to be quite effective for feature selection and classification accuracy improvement. All these researches related to GA are data specific or algorithm specific. In [22] the performance of GA is compared with other feature reduction and extraction techniques like liner discriminant analysis (LDA); principle component analysis (PCA) for one dataset GA was found to perform better while for the other dataset LDA and PCA showed promising results. In [23] the genetic algorithm is combined with the boosting technique in order to improve accuracy of classification. The improved version assigns higher weight to the misclassified instances in order to shift the focus on them in the next iteration. This process tends to achieve higher accuracy with less number of evaluations than the original GA. In [24] genetic algorithm is implemented in combination with K-nearest neighbor classifier and feature extraction; reduction and classifier training are all done simultaneously and results are compared with other industry standard feature extraction and reduction technique like liner discriminant analysis and sequential floating forward feature selection.

Despite all this extensive work on ensemble methods and feature reduction problem and various classification algorithms for improving the accuracy rate in classification, there is no global optimization ensemble model suggested so far that can improve the accuracy of classification methods with any dataset. Therefore in this paper we design and implement such a global optimization model.

## 3. Design

**Figure 1:**
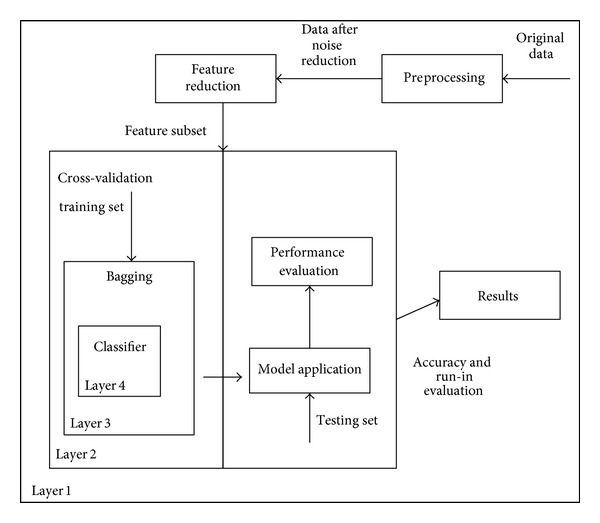
Design of global optimization ensemble model for classification methods (GMC).

The idea was to implement the concept of ensemble model in order to create an improved global model.  Figure 1 shows the overall design of the ensemble model.

Layer 1 was providing antidote for dimensionality curse. As discussed in the literature review the dimensionality reduction or feature reduction is necessary in order to improve the classification accuracy. Therefore in our model the first layer contains the data set, preprocessing operator, and a feature reduction operator. For feature selection, genetic algorithm (GA) is used as it has shown to produce better results than other feature reduction techniques [23–25].

Maximal fitness of GA is set to infinity as there is no absolute maxima for the fitness function which means the GA will keep on selecting the best of best until the stop criteria are met which in this case is the maximum number of generation. Roulette wheel selection scheme was used for selecting individuals because it has the obvious advantage that it does not ignore or discard any individuals and each individual is given a chance of being chosen as even the weakest of individuals might be hiding valuable information. And as we are striving for a global solution, therefore a selection method that preserves diversity and is fast to converge sounds good. Crossover type was set to shuffle because shuffle crossover is related to uniform crossover. A single crossover position (as in single-point crossover) is selected. But before the variables are exchanged, they are randomly shuffled in both parents. After recombination, the variables in the offspring are unshuffled. This removes positional bias as the variables are randomly reassigned each time crossover is performed. The parameter values chosen for GA are shown in Table 1.

Parameter optimization for the operators in each layer was done by implementing global optimization operator using grid search. This methodology involves setting up of grids in the decision space and evaluating the values of the objective function at each grid point. The point which corresponds to the best value of the objective function is considered to be the optimum solution. For all the layers, a total of 5 parameters were optimized using grid search optimizations. From each attribute 11 combinations were proposed; this means for optimizing these 5 parameters total 161051 combinations were tested.  Table 2(a) shows all the parameter and there optimized values.

In layer 2 partition of training and testing dataset was done using X-fold crossvalidation. The data set is divided into *n* subsets, and the holdout method is repeated *k* times. Each time, one of the *n* subsets is used as the test set and the other *n* − 1 subsets are put together to form a training set. Then the average error across all *n* trials is computed. The advantage of this method was that it matters less how the data gets divided. Every data point gets to be in a test set exactly once and gets to be in a training set *n* − 1 times. Besides, the variance of the resulting estimate is also reduced as *n* is increased. Stratified sampling scheme was used in CV with number of iteration set to 10 as shown in Table 2(a). In stratified sampling the random subsets are created but the distribution of class in those subsets is the same as the whole dataset. Thus this type of sampling reduces variance. For example we have a data set of 180 employees and we want a sample set of 40 employees. The first step is to calculate the percentage of male female in each group, that is,

- percentage of male members in full-time category = 90/180 = 50%,
- percentage of male members in part-time category = 18/180 = 10%,
- percentage of female members in full-time category = 9/180 = 5%,
- percentage of female members in part-time category= 63/180 = 35%.

This calculation tell us that of our desired sample of 40 employees, 50 percent should be male (full time), 10 percent should be male (part-time), 5 percent should be female (full-time), and 35 percent should be female (part-time). This means that we need to calculate the 50% of 40 which is 20. Similarly 10% of 40 is 4, 5% of 40 is 2, and 35% of 40 is 14. This is the final ratio of records in each category in our sample of 40 employees.

Layer 3 did an optimal bias-variance tradeoff. Accuracy improvement is done by implementing bootstrap aggregation (bagging). Bagging is a machine learning ensemble meta-algorithm which reduces both bias and variance in order to help avoid overfitting. Although it is usually applied to decision tree models, it can be used with any type of model. Bagging is a special case of the model averaging approach. Parameter setting for bagging is shown in Table 2(a). We are using bagging instead of boosting because error = noise error + bias + variance bagging can reduce both bias and variance but mostly it reduces just variance and it hardly ever increases error. For high-bias classifiers, it can reduce bias and for high-variance classifiers, it can reduce variance, while boosting in the early iterations is primary a bias-reducing method. In later iterations, it appears to be primarily a variance-reducing method. It may increase error and margins and is not good with data with noise. That is the reason why we chose bagging instead of boosting for bias and variance tradeoff.

Classifiers were placed in layer 4 with parameters configuration done according to the dataset. All classifier parameters were set to obtain the optimal model in order to reduce the bias. The setting used for each classifier is shown in Table 2(b).

## 4. Implementation

Implementation and testing are done using core i3 processor with 4 GB RAM, while coding is done using XML. Preprocessing is performed on every dataset according to requirements of the classifier used in order to remove noise from data and do type conversations. The model is implemented and tested in RapidMiner5 [15].

**Step 1 (algorithm selection)**

As we are optimizing the model for supervised learning problems, therefore the following liner and nonliner classifiers were selected, implemented, and tested.

**Step 2 (data set selection)**

Datasets from various different fields are selected such as banking, medicine, and census data. Selection of the datasets was based on

- relevant to classifier,
- frequency of citations,
- a wide range of values in terms of attributes classes and attribute characteristic.

In total 7 datasets from various fields are used for experimentation. The classifiers used in the implementation and the details of the datasets used are given in Tables 3 and 4 respectively.

Suitable classifier for each dataset is selected and indicated as shown in Table 5.

**Step 3 (simple classification using validation technique)**

First each dataset is classified using the classifier mentioned for each dataset and the results are validated using the X-fold cross-validation technique where *x* = 10 for all classifiers. Sampling technique used for validation is “stratified sampling.”

Results consisting of classification accuracy and classification error are recorded for each classifier.

**Step 4 (classification using global optimization ensemble model for classification methods (GMC))**

All the classifiers are now encapsulated in the proposed generic optimization ensemble model and executed for results. Parameters of all the classifiers are the same as in Step 3. Now the improved results consisting of optimized classification accuracy are recorded for every classifier and compared with the previous result in order to calculate the improvement percentage.

## 5. Results

The results for each data set and the corresponding accuracy comparison between simple classification and GMC model are given in this section.

Table 6 shows that using the GMC model for optimizing the classification accuracy for cancer dataset has improved from 1.13% to 29.76% depending on the classifier and the bias-variance tradeoff its inner complexity offers.

Table 7 shows that using the GMC model for optimizing the classification accuracy for heart disease dataset has improved from 2.4% to 14.54% depending on the classifier and the bias-variance tradeoff its inner complexity offers.

Table 8 shows that using the GMC model for optimizing the classification accuracy for wine dataset has improved from 3.92% to 19.67% depending on the classifier and the bias-variance tradeoff its inner complexity offers.

As shown in Table 9, using the GMC model for optimizing the classification accuracy for adult income dataset has improved from 1% to 6.5% depending on the classifier and the bias-variance tradeoff its inner complexity offers.

As shown in Table 10, using the GMC model for optimizing the classification accuracy for sonar dataset has improved from 4.82% to 15.36% depending on the classifier and the bias-variance tradeoff its inner complexity offers.

As shown in Table 11, using the GMC model for optimizing the classification accuracy for educational dataset has improved from 8% to 26% depending on the classifier and the bias-variance tradeoff its inner complexity offers.

As shown in Table 12, using the GMC model for optimizing the classification accuracy for diabetes dataset has improved from 1.39% to 10.55% depending on the classifier and the bias-variance tradeoff its inner complexity offers.

It can be seen that for K-NN the improvement in accuracy is as high as 30%. This is a significant increase in accuracy and shows the effectiveness of the GMC model. For other algorithms such as decision tress or logistic regression the increase in classification accuracy is varying between 1% and 3%. Although this may indicate the shorting comings of GMC model, but this is not the case in reality. During the experimentation it was noted that for some supervised techniques such as decision tress, the accuracy of the classifier was already very high (90% or more); therefore the possibility of further improving the classifier was rather limited. Thus in this case GMC model could only increase the accuracy by a small amount. For example in the case of decision tress, the GMC model increased the average accuracy from 91.1% to 93.2%. However for other supervised learning algorithms there existed a large gap within which the classifier accuracy may further be increased. This explains the significant increase classifier accuracy in algorithms such as K-NN. Thus although GMC model is dependent upon the algorithm in terms of how much classifier accuracy may be improved, yet it has in all cases increased the accuracy of the classifier.

## 6. Conclusion and Further Work

In order to solve the basic issues of supervised learning problems like dimensionality reduction, bias-variance tradeoff, and noise, we used the concept of ensemble models to design an optimized global ensemble model for classification methods (GMC). The model was designed in layers with each layer solving one of the basic issues of supervised learning. We proved through experimentation that if classifiers are enclosed in our model there accuracy improves from 1% to 30% depending upon the algorithm complexity and its capability of handling bias and variance. Our model yielded better results than when the classifiers were used alone or in combination.

The model can be further optimized for extremely large data set in real time. In that case the optimization will focus on the reduction of execution time as well as further improvement in accuracy. Parallel processing can be introduced into the model for minimizing time. There are a lot of optimization techniques available and a separate research and comparison can be carried out between all those techniques and the effect of those techniques on the global model. Furthermore, research can be carried out on this model for unsupervised learning problems with data sets related to more diverse fields.

## Figures and Tables

**Table 1:** Parameter values in GA.

Parameter	Value
Selection scheme	Roulette wheel
Crossover type	shuffle
Probability of crossover	0.5
Probability for initial population	0.5
Probability of mutation	1/number of attribute
Maximal fitness	Infinity

**Table:** (a)

Parameter	Operator	Grid range	Combination	Scale	Optimal value
Population size	GA-layer 1	2–100	2, 3, 6, 11, 18, 27, 37, 50, 65, 81, 100	Quadratic	6
Maximum number of generation	GA-layer 1	1–50	1, 6, 11, 16, 21, 26, 30, 35, 40, 45, 50	Linear	16
Number of iterations	CV-layer 2	2–50	2, 4, 6, 10, 14, 19, 26, 33, 41, 50	Quadratic	10
Sampling Size	Bagging-layer 3	0–1.0	0, 0.1, 0.2, 0.3, 0.4, 0.5, 0.6, 0.7, 0.8, 0.9, 1	Linear	0.6
Number of iterations	Bagging-layer 3	1–100	1, 2, 5, 10, 17, 26, 37, 50, 64, 81, 100	Quadratic	10

**Table:** (b)

Operator name	Parameter configuration
ID 3	Criterion: information_gain
	Minimal size of split: 4
	Minimal leaf size: 2
	Minimal gain: 0.1

Decision tree	Criterion: Information_gain
	Minimal size for split: 4
	Minimal leaf size: 2
	Minimal gain: 0.1
	Maximal depth: 20
	Confidence: 0.5

Random forest	Number of trees: 10
	Criterion: Information_gain
	Minimal leaf size: 2
	Minimal gain: 0.1
	Maximum depth: 20
	Confidence: 0.5

Rule induction	Criterion: information_gain
	Sample ratio: 0.7
	Pureness: 0.6
	Minimal prune benefit: 0.6

K-NN	K nearest neighbors: 11
	Weighted vote: true
	Measure type: nominal measures
	Nominal measure: Dice similarity

Naïve bayes	Laplace correction: true

W-AODE	Frequency for super parents: 1.0

W-PART	Confidence threshold: 0.5
	Minimum objects per leaf: 2.0

W-J48	Confidence threshold: 0.5
	Minimum objects per leaf: 2.0

**Table 3:** Classifiers.

Classifiers	
KNN [4, 5]	
Decision tree [6, 7]	
ID3 [8, 9]	
Random forest [10, 11]	
Logistic regression [12, 13],	
Rule induction [14]	
W-AODE, W-PART, W-Prism, W-J48 [15]	

**Table 4:** Data set details.

Data set	Number of cases	Number of attribute	Number of classes	Attribute characteristics	Missing values
Cancer dataset	699	9	2	Numeric	Yes
Diabetes dataset	768	9	2	Integer and real	No
Heart disease dataset	303	14	2	Categorical, integer, and real	Yes
Adult income dataset	1000	15	2	Integer and nominal	No
Wine dataset	178	13	3	Real and integer	No
Sonar dataset	208	61	2	Real and nominal	yes
Educational progress dataset	50	9	3	Nominal	No

**Table 5:** Data set and suitable classifiers.

Dataset	Classifier	Capabilities
All datasets	K-NN	Polynomial, numerical, binomial attributes, and labels can handle missing values

All datasets	Decision tree	Polynomial, numerical, and binomial attributes cannot handle numeric labels and can handle missing values
Heart, wine, and educational and sonar dataset	Rule induction	

Cancer, heart, adult income dataset	ID3	Can only handle binomial and polynomial labels and attributes and cannot handle missing values
All datasets	W-AODE	
All datasets	W-Prism	

Educational progress and sonar and adult income dataset	Random forest	Polynomial, numerical, and binomial attributes cannot handle numeric labels and cannot handle missing values
All datasets	W-PART	
All datasets	W-J48	

Sonar, diabetes, cancer, andadult income dataset	Logistic regression	Numerical attributes and binomial labels cannot handle missing values

**Table 6:** Results for cancer dataset: comparison of optimized classification accuracy using GMC model with simple classification using different classifiers.

Algorithm	Classification accuracy	Optimized classification accuracy	Improvement %
K-NN	66.81%	96.57%	**29.76%**
Decision tree	94.42%	96.71%	**2.29%**
ID3	66.52%	85.27%	**18.52%**
W-PART	94.71%	97.28%	**2.57%**
W-Prism	90.13%	96.28%	**6.15%**
W-J48	94.71%	96.71%	**2%**
W-AODE	97.00%	100%	**3%**
Logistic regression	95.01%	96.14%	**1.13%**

**Table 7:** Results for heart disease dataset: comparison of optimized classification accuracy using GMC model with simple classification using different classifiers.

Algorithm	Classification accuracy	Optimized classification accuracy	Improvement %
K-NN	50.82%	59.75%	8.93%
Decision tree	44.89%	59.43%	14.54%
ID3	47.52%	55.48%	8.24%
W-PART	50.52%	60.08%	9.56%
W-Prism	47.51%	56.09%	8.58%
W-AODE	55.47%	61.13%	5.66%
W-J48	49.87%	61.05%	11.18%
Rule induction	57.72%	59.76%	2.4%

**Table 8:** Results of wine dataset: comparison of optimized classification accuracy using GMC model with simple classification using different classifiers.

Algorithm	Classification accuracy	Optimized classification accuracy	Improvement %
K-NN	70.75%	90.42%	**19.67% **
Decision tree	91.57%	95.49%	**3.92% **
W-PART	90.42%	96.67%	**6.25% **
W-Prism	52.32%	61.27%	**8.95% **
W-AODE	71.34%	75.26%	**3.92% **
W-J48	90.46%	96.63%	**6.17% **
Rule induction	86.37%	93.27%	**6.9% **

**Table 9:** Results of adult income dataset: comparison of optimized classification accuracy using GMC model with simple classification using different classifiers.

Algorithm	Classification accuracy	Optimized classification accuracy	Improvement %
K-NN	76.70%	83.20%	**6.5%**
Decision tree	80.00%	82.20%	**2.20%**
ID3	75.60%	78.60%	**3%**
W-PART	81.00%	83.50%	**2.4%**
W-Prism	81.10%	82.20%	**1.1%**
W-AODE	80.80%	82.60%	**1.8%**
W-J48	81.50%	83.00%	**1.5%**
Random forest	76.10%	77.30%	**1.2%**
Logistic regression	79.00%	80.00%	**1%**

**Table 10:** Results of sonar dataset: comparison of optimized classification accuracy using GMC model with simple classification using different classifiers.

Algorithm	Classification accuracy	Optimized classification accuracy	Improvement %
K-NN	69.71%	74.57%	**4.86%**
Decision Tree	73.57%	83.67%	**10.1%**
W-PART	75.48%	83.17%	**7.69%**
W-Prism	48.02%	63.38%	**15.36%**
W-J48	70.24%	82.21%	**11.97%**
Rule induction	71.66%	76.48%	**4.82%**
Random forest	68.26%	75.36%	**7.1%**
Logistic regression	74.55%	80.29%	**5.74%**

**Table 11:** Results of educational dataset: comparison of optimized classification accuracy using GMC model with simple classification using different classifiers.

Algorithm	Classification accuracy	Optimized classification accuracy	Improvement %
K-NN	46%	54%	**8% **
Decision Tree	42%	56%	**14% **
ID3	20%	44%	**24% **
W-PART	32%	54%	**22% **
W-Prism	24%	50%	**26% **
W-J48	44%	58%	**14% **
W-AODE	46%	56%	**10% **
SVM	60%	76%	**16% **
Random forest	48%	58%	**12% **
Rule induction	44%	54%	**10% **

**Table 12:** Results of diabetes dataset: comparison of optimized classification accuracy using GMC model with simple classification using different classifiers.

Algorithm	Classification accuracy	Optimized classification accuracy	Improvement %
K-NN	73.70%	77.48%	**4% **
Decision tree	74.0%	75.39%	**1.39% **
W-PART	73.83%	77.34%	**3.51% **
W-Prism	57.42%	67.97%	**10.55% **
W-J48	74.08%	77.22%	**3.14% **
W-AODE	66.54%	69.14%	**2.6% **
Logistic regression	76.00%	77.95%	**1.95% **

## References

[1] Han J, Kamber M (2006). *Data Mining: Concepts & Techniques*.

[2] Han J, Kamber M, Pei J (2005). *Data Mining: Concepts and Techniques*.

[3] Kotsiantis S, Kanellopoulos D, Pintelas P (2006). Data preprocessing for supervised leaning. *International Journal of Computer Science*.

[4] Toussaint G (2005). Geometric proximity graphs for improving nearest neighbor methods in instance-based learning and data mining. *International Journal of Computational Geometry and Applications*.

[5] Söder O http://www.fon.hum.uva.nl/praat/manual/kNN_classifiers_1__What_is_a_kNN_classifier_.html.

[6] Safavian SR, Landgrebe D (1991). A survey of decision tree classifier methodology. *IEEE Transactions on Systems, Man and Cybernetics*.

[7] Li J http://sites.stat.psu.edu/~jiali/course/stat597e/notes2/trees.pdf.

[8] Wang H, Wang L, Yi L Maximum entropy framework used in text classification.

[9] Mitchell TM (1997). *Machine Learning*.

[10] Breiman L (2001). Random forests. *Machine Learning*.

[11] Ho TK Random decision forest.

[12] Hosmer DW, Lemeshow S (2000). *Applied Logistic Regression*.

[13] Cohen J, Cohen P, West SG, Aiken LS (2002). *Applied Multiple Regression/Correlation Analysis For the Behavioral Sciences*.

[14] Quinlan JR, McDermott J Generating production rules from decision trees.

[15] http://www.rapid-i.com/downloads/tutorial/rapidminer-4.6-tutorial.pdf.

[16] Dash S, Patra B, Tripathy BK (2012). A hybrid data mining technique for improving the classification accuracy of microarray data set. *International Journal of Information Engineering and Electronic Business*.

[17] Lin S-W, Chen S-C (2009). PSOLDA: a particle swarm optimization approach for enhancing classification accuracy rate of linear discriminant analysis. *Applied Soft Computing Journal*.

[18] Bryll R, Gutierrez-Osuna R, Quek F (2003). Attribute bagging: improving accuracy of classifier ensembles by using random feature subsets. *Pattern Recognition*.

[19] Abbott DW Combining models to improve classifier accuracy and robustness.

[20] Sohn SY, Lee SH (2003). Data fusion, ensemble and clustering to improve the classification accuracy for the severity of road traffic accidents in Korea. *Safety Science*.

[21] Smith MR, Martinez T Improving classification accuracy by identifying and removing instances that should be misclassified.

[22] Zamalloayz M, Rodriguez-Fuentesy LJ, Penagarikanoy M, Bordely G, Uribez JP Feature dimensionality reduction through genetic algorithms for faster speaker recognition.

[23] Liu B, McKay B, Abbass HA Improving genetic classifiers with a boosting algorithm.

[24] Raymer ML, Punch WF, Goodman ED, Kuhn LA, Jain AK (2000). Dimensionality reduction using genetic algorithms. *IEEE Transactions on Evolutionary Computation*.

[25] Kumar DN Optimization Methods: Advanced Topics in Optimization—Direct and Indirect Search Methods. http://www.gninagpur.in/nptel/courses/Webcourse-contents/IISc-BANG/OPTIMIZATION%20METHODS/pdf/Module_8/M8L4_LN.pdf.

